# Primary pleomorphic rhabdomyosarcoma of the adrenal gland in an adult: A case report

**DOI:** 10.3892/ol.2013.1674

**Published:** 2013-11-11

**Authors:** CHAO-JUN WANG, JUN LI, JIE QIN

**Affiliations:** 1 Department of Urology, The First Affiliated Hospital, College of Medicine, Zhejiang University, Hangzhou, Zhejiang 310003, P.R. China; 2 Department of Pathology, The First Affiliated Hospital, College of Medicine, Zhejiang University, Hangzhou, Zhejiang 310003, P.R. China

**Keywords:** rhabdomyosarcoma, adrenal, adult

## Abstract

A 61-year-old female was referred to The First Affiliated Hospital, College of Medicine, Zhejiang University (Hangzhou, China) due to a right adrenal tumor. A pre-operative transcutaneous fine-needle aspiration biopsy and right adrenalectomy were performed, and pathological analysis resulted in the diagnosis of pleomorphic rhabdomyosarcoma (RMS). Primary pleomorphic RMS of the adrenal gland in an adult is a rare condition. To the best of our knowledge, this is the first case of pleomorphic RMS of the adrenal gland in an adult diagnosed by light microscopy and immunohistochemical stains.

## Introduction

Rhabdomyosarcoma (RMS) is a malignant soft-tissue sarcoma that is believed to develop from primitive totipotent embryonic mesenchyme. RMS is a highly aggressive tumor with a tendency for advanced and disseminated disease early in its course. The condition is the most common soft tissue sarcoma in children. However, RMS in adults is an uncommon tumor that arises mainly in the large skeletal muscles (1–4). Pleomorphic RMS was first described by Stout in 1946 (5). More recent studies have reported that pleomorphic RMS is rare and occurs predominantly in adults. The present study describes a case of pleomorphic RMS in the right adrenal region of a 61-year-old female and reviews the literature on this rare disease. The study was approved by the ethics committee of the First Affiliated Hospital, School of Medicine, Zhejiang University (Hangzhou, China). Informed consent was obtained from the patient.

## Case report

**Figure 1:**
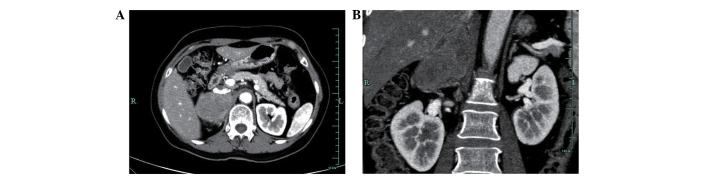
(A) Axial contrast-enhanced computed tomography (CT) scan showing the right adrenal tumor. (B) CT coronal section showing the right adrenal tumor.

A 61-year-old female was referred to the urological ward of The First Affiliated Hospital, College of Medicine, Zhejiang University for a right adrenal mass that had been detected incidentally by ultrasound examination two weeks previously. The patient had no underlying disease and the physical examination was unremarkable. Abdominal ultrasound revealed a large adrenal tumor. A computed tomography scan revealed a right adrenal tumor measuring 6.0×4.0 cm (Fig. 1). The patient had undergone a complete adrenal endocrinological evaluation, which demonstrated that the lesion was not a secreting tumor.

**Figure 2:**
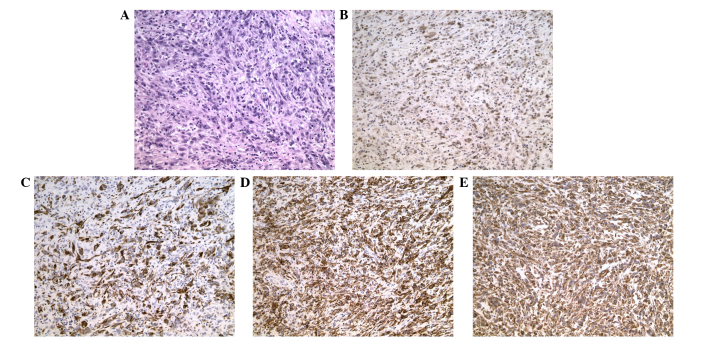
Pathological examination showing pleomorphic rhabdomyosarcoma. (A) Hematoxylin and eosin staining. Spindle-shaped cells arranged in a fascicular pattern were present. (B) Immunohistochemical staining showing positivity for (B) MyoD1, (C) desmin, (D) CD56 and (E) vimentin. Magnification, ×200.

A pre-operative transcutaneous fine-needle aspiration biopsy was performed and the cytological diagnosis was consistent with a malignant neoplasm. Right adrenalectomy was performed. The tumor was observed to have invaded into the right lobe of the liver, and was well-demarcated from the hepatic parenchyma by a thick fibrous capsule. The total operating time was 3 h. The estimated blood loss was 200 ml (calculated and recorded by the attending anesthetist). The patient tolerated the procedure well and there were no post-operative complications. The drainage tube was removed at 48 h following the surgery. The patient was discharged on the fifth post-operative day, tolerating a regular diet. Pathological examination of the surgical specimen was pleomorphic RMS containing spindle cells (Fig. 2A). Immunohistochemistry revealed a positive stain for MyoD1, desmin, vimentin and CD56 (Fig. 2B–D). No expression of smooth muscle actin (SMA), SYN or S100 protein was identified in the tumor tissue.

## Discussion

In the current World Health Organization Classification of Soft Tissue and Bone Neoplasms, RMS is divided into three distinct subtypes, embryonic, alveolar and pleomorphic (6). RMS is a rare disease of the adrenal gland neoplasm, which predominantly occurs in adults. Charytonowicz *et al*(7) suggest that RMS may arise from non-muscle cells, including mesenchymal stem cells. Theoretically, RMS may affect any body part, including the adrenal glands, as shown in the present case. To date, only two RMS cases in the adrenal region have been described in the English literature. Yi *et al*(8) reported a case of alveolar RMS in the right adrenal region of a pediatric patient with a characteristic history of hypertension and fever. Katayama *et al*(9) reported a case of RMS in the adrenal region of an elderly hypertensive patient. However, pleomorphic RMS of the adrenal gland in an adult has not been previously reported.

In the present case, light microscopic examination revealed a malignant pleomorphic mesenchymal neoplasm, characterized mainly by the proliferation of atypical spindle cells and few epithelioid cells. Immunohistochemistry revealed positive staining for MyoD1, desmin, vimentin and CD56. By contrast, no expression of SMA, SYN or S100 protein was identified in the tumor tissue. A diagnosis of pleomorphic RMS was confirmed according to the clinical and pathological findings.

In conclusion, the present study described a rare case of pleomorphic RMS in the right adrenal region based on the histopathology and immunohistochemistry results. Due to the small number of described cases of adrenal gland RMS, inadequate information is available for evaluating the treatment procedure and the final prognosis of the patient. An accumulation of such cases and an improved understanding of the molecular biology driving RMS tumor behavior are required for further evaluation and research to identify the histogenesis of the condition. Primary pleomorphic RMS of the adrenal gland in an adult is a rare condition. To the best of our knowledge, this is the first case of pleomorphic RMS of the adrenal gland in an adult diagnosed by light microscopy and immunohistochemical staining.
